# 3D Morphology, Ultrastructure and Development of *Ceratomyxa puntazzi* Stages: First Insights into the Mechanisms of Motility and Budding in the Myxozoa

**DOI:** 10.1371/journal.pone.0032679

**Published:** 2012-02-29

**Authors:** Gema Alama-Bermejo, James Emmanuel Bron, Juan Antonio Raga, Astrid Sibylle Holzer

**Affiliations:** 1 Marine Zoology Unit, Zoology Deparment, Cavanilles Institute of Biodiversity and Evolutionary Biology, University of Valencia, Paterna, Valencia, Spain; 2 Parasitology group, Institute of Aquaculture, University of Stirling, Stirling, United Kingdom; 3 Laboratory of Fish Protistology, Institute of Parasitology, Biology Centre, Academy of Sciences of the Czech Republic, Ceske Budejovice, Czech Republic; University of Colorado, Boulder, United States of America

## Abstract

Free, amoeboid movement of organisms within media as well as substrate-dependent cellular crawling processes of cells and organisms require an actin cytoskeleton. This system is also involved in the cytokinetic processes of all eukaryotic cells. Myxozoan parasites are known for the disease they cause in economical important fishes. Usually, their pathology is related to rapid proliferation in the host. However, the sequences of their development are still poorly understood, especially with regard to pre-sporogonic proliferation mechanisms. The present work employs light microscopy (LM), electron microscopy (SEM, TEM) and confocal laser scanning microscopy (CLSM) in combination with specific stains (Nile Red, DAPI, Phalloidin), to study the three-dimensional morphology, motility, ultrastructure and cellular composition of *Ceratomyxa puntazzi*, a myxozoan inhabiting the bile of the sharpsnout seabream.

Our results demonstrate the occurrence of two *C. puntazzi* developmental cycles in the bile, i.e. pre-sporogonic proliferation including frequent budding as well as sporogony, resulting in the formation of durable spore stages and we provide unique details on the ultrastructure and the developmental sequence of bile inhabiting myxozoans. The present study describes, for the first time, the cellular components and mechanisms involved in the motility of myxozoan proliferative stages, and reveals how the same elements are implicated in the processes of budding and cytokinesis in the Myxozoa. We demonstrate that F-actin rich cytoskeletal elements polarize at one end of the parasites and in the filopodia which are rapidly *de novo* created and re-absorbed, thus facilitating unidirectional parasite motility in the bile. We furthermore discover the myxozoan mechanism of budding as an active, polarization process of cytokinesis, which is independent from a contractile ring and thus differs from the mechanism, generally observed in eurkaryotic cells. We hereby demonstrate that CLSM is a powerful tool for myxozoan research with a great potential for exploitation, and we strongly recommend its future use in combination with *in vivo* stains.

## Introduction

Most animals are motile. Three main types of animal movement can be differentiated, movement via skeletal muscles, via cilia and flagella, and amoeboid movement or cellular crawling. Amoeboid movement is typical of amoebae and unicellular organisms, but also of metazoan cells like leukocytes [1]. The motility mechanisms in all cells rely on key molecular components functionally conserved from protozoans to vertebrates [2]. The machinery that powers cell migration is built from the actin cytoskeleton, and amoeboid movement is generally accepted to be based on a cytoskeleton which allows membrane protrusion [3]. Protrusion or forward motility is based on the extension of pseudopodia that can be of three kinds: filopodia, lamellipodia or blebs [4]. Filopodia and lamellipodia are produced by polymerization of actin, but blebs are membrane bulgings that are actomyosin-dependent [5].

In eukaryotes, the actin system also provides the force for cell divisions, representing the key in the process of division of one cell into two by the formation of a contractile ring during cytokinesis [6]. Animal cytokinesis is explained by the purse-string model or cytokinesis A, *i.e.* a contractile ring composed of actin and myosin II that drives the equatorial furrowing [7], and it has been demonstrated that filamentous actin (F-actin) polymerization is important for the assembly, maintenance and closure of the contractile ring between two cells [6]. However, other cytokinetic modes have been observed in animal cells: Cytokinesis B is an attachment-assisted cleavage whereas cytokinesis C is a traction-mediated cytofission of multinucleate cells [7]–[11]. Both modes are driven by actin polymerization [7], [12]–[14]. Motility and cytokinetic mechanisms based on actin polymerization appear to be more primitive than those based on ATPase motor proteins (myosins, dyneins and kinesins) [3]. Thus, cytokinesis B and C seem to be primitive methods of division of eukaryotic cells, while cytokinesis A, is functionally more evolved [11].

The study of cytokinesis in parasites has mainly been centred in protozoans revealing unusual mechanisms of cytokinesis, *e.g. Giardia intestinalis* [15] or *Trypanosoma* [16].

The Myxozoa is an economically important group of microscopic metazoan endoparasites. Myxozoans have an indirect life cycle involving an invertebrate host, usually an annelid, and a vertebrate host, usually a teleost fish. In both hosts, the parasite proliferates and then forms durable spore stages that infect the other host. A number of species within the phylum Myxozoa are recognised as important pathogens of commercially exploited wild and cultured finfish [17]–[20]. Pathology in myxozoans is usually associated with the presence of large numbers of parasites, produced by rapid proliferation of vegetative stages [21]–[22]. However, little is known about the developmental sequences and the cytokinetics resulting in the multiplication of myxozoan parasite stages.

Members of the myxozoan genus *Ceratomyxa* generally inhabit the bile and have been reported to show motility and amoebic movement [23]–[26]. Amoebic movement has also been reported from sporoplasms after release from the spore, *e.g.* in *T. bryosalmonae* [27] and *M. cerebralis* [28]–[29].While motility of the sporoplasms allows the parasite to burrow into the epithelia of the fish, thus allowing entry into or, later on, active displacement within the host's tissues, motility in myxozoans inhabiting body cavities filled with fluids probably serves suspension rather than displacement. As myxozoans occurring in the bile are often present in large numbers and can be easily extracted without host tissue contamination, they provide an excellent opportunity to study the structures facilitating parasite motility and the mechanisms underlying parasite movement, in order to fill this important gap in our knowledge of this parasite group.

Traditionally, light microscopy and electron microscopy are being used to describe the morphology of different stages. In 2005, McGurk et al. [30] published the first three-dimensional view of myxozoans by studying spores of *T. bryosalmonae* using confocal laser scanning microscopy. This powerful technique allows for the visualization of different cell components in whole parasites, with minimal processing of the material and a wide range of fluorescent dyes available for the visualization of different morphological features [30], and is awaiting further exploitation.

We recently found large numbers of different developmental stages of *Ceratomyxa puntazzi* in the bile of the sharpsnout seabream *Diplodus puntazzo* [31]. These parasite stages showed strong motility and a high rate of cell proliferation. In this paper we combine a variety of microscopic techniques, *i.e.* light microscopy, scanning and transmission electron microscopy as well as confocal laser microscopy, to study different *C. puntazzi* development stages' in order to elucidate their three-dimensional morphology, ultrastructure and composition, and to better understand the mechanisms underlying myxozoan locomotion and the structural components allowing motility and cytokinesis in this parasite group. Furthermore, we aim to ascertain the sequence of parasite development resulting in successful proliferation and spore formation.

## Results

We found a range of different stages of *C. puntazzi* in the bile of *D. puntazzo*. Most importantly, two different developmental cycles of the parasite were observed: 1. Pre-sporogonic proliferative development and 2. Sporogony. Both developmental cycles were found to occur in parallel but fish were observed to have either predominantly stages lacking mature spores or predominantly stages with mature spores.

### Phalloidin staining reveals the distribution and function of F-actin in myxozoan stages during motility and budding

**Figure 1:**
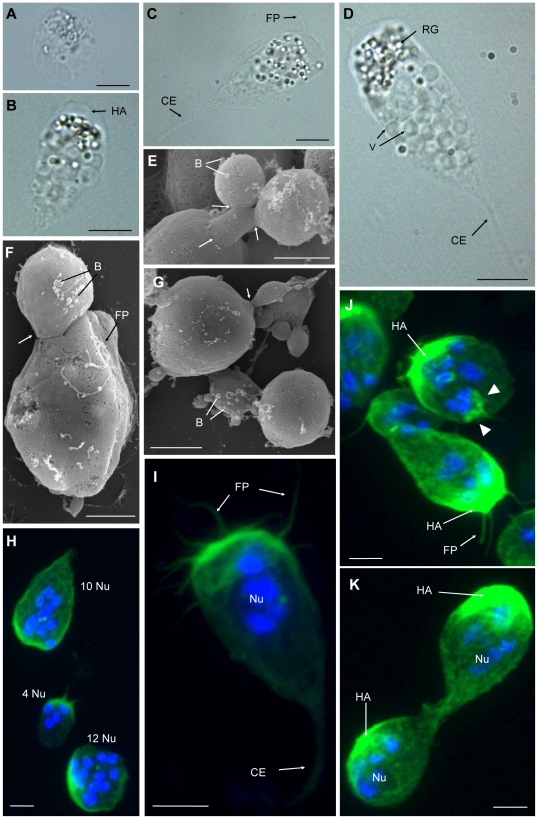
Motility and budding of *Ceratomyxa puntazzi* in the bile of *Diplodus puntazzo*. A–D: LM, E–G: SEM, H–K: CLSM (DAPI and Phalloidin stained). A) Small ellipsoidal stage. B) Pyriform stage with a wide hyaline area and refractive granules at rounded, anterior end of parasite. C) Pyriform stage showing large filopodia and abundant refractive bodies at rounded end and a large, rigid cytoplasm extension at posterior end. D) Pyriform stage with abundant vacuoles present in almost the whole body. Refractive bodies were concentrated at anterior end. E–G) Exogenous budding with several stages dividing by plasmotomy. Arrows indicate cytoplasm constrictions. Some filopodia and blebs can be seen on the surface of the stages. H) Three stages, a small ellipsoidal stage with 4 nuclei and two larger stages with 10 and 12 nuclei. I) Pyriform stage with abundant filopodia at round side, where F-actin (green stain) is accumulated, and rigid cytoplasmic extension at the posterior end. Four nuclei are visible. J) Several stages with a clear pattern of accumulation of F-actin in the hyaline area at the anterior end of the parasites where the filopodia are located. Upper parasite: exogenous budding of a round stage with three nuclei (arrow head) and an F-actin rich surface at opposite end from the “mother” parasite it is emerging from. K) Two stages showing exogenous budding with still attached buds moving in opposite directions. Abbreviations: HA: hyaline area; CE: cytoplasmic extension; FP: filopodia; RG: refractive granules; V: vacuole; B: bleb; Nu: Nuclei. Scale Bar: A = 3 µm; B–D = 10 µm; E–K = 4 µm.

**Figure 2:**
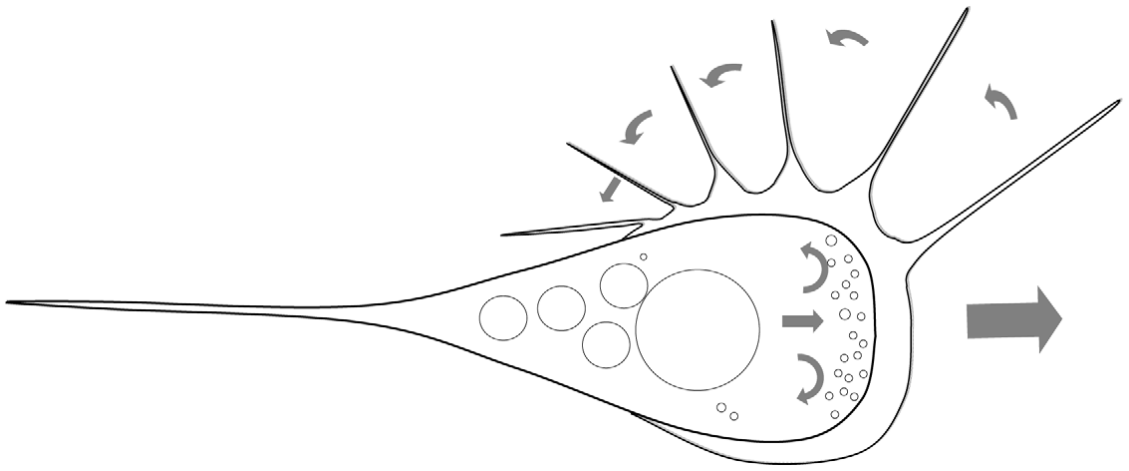
Schematic drawing of the locomotive action of an active pyriform stage. Projection of filopodia from the anterior, median part radially to most posterior part of the hyaline area, allowing active parasite movement.

Proliferating stages presented high morphological plasticity and locomotive activity, showing amoeboid movement. The earliest and smallest stages were round or ellipsoidal (Figure 1A), measured 3.7–6.5 µm in diameter, and showed dispersed refractive bodies in their cytoplasm as well as the presence of 1–3 filopodia of 0.9–3.1 µm length. As development progressed, the parasites acquired a larger size (9.7–35.2 µm length, 5.89–20.52 µm width; 3–11 filopodia of 1.7–7.5 µm length) and a characteristic pyriform shape, which seemed to be closely associated with the direction of movement (Figure 1B–1D). Thereby, the round side was found to represent the anterior end of parasites in motion, and it was characterized by a hyaline area (ectoplasm) of 2.3–3.5 µm width and a concentration of filopodia which were moving actively, and were sometimes ramified (Figure 1B and 1I; Video S1). Using CLSM, a high concentration of F-actin was detected at the round, anterior end of the parasites (green phalloidin staining in Figure 1H–K), corresponding to the hyaline ectoplasm area and in the filopodia. The posterior end of the parasites was typically pointed, forming a single, large cytoplasmic extension (8.1–13.9 µm length, 1.1–2 µm and 0.3–1.3 µm width at base and at the tip, respectively) which had a much more rigid appearance than the delicate filopodia at the anterior end of the parasites, and which lacked accumulation of F-actin (Figure 1C–D and 1I). We could observe an undulating motion of the hyaline area *in vivo*, probably provoked by the projection of the filopodia, which were formed at the anterior end of the parasites and merged with the cell surface in the lateral, posterior area (Video S1). The filopodia were projected in the direction of parasite movement and then posteriorly, from the median anterior end, which usually had the largest filopodia, to the most posterior part of the body. Filopodia movement was substrate-independent and occurred radially around the whole parasite. Thereby the parasites rowed themselves forward in the medium (Figure 2; Video S1). In the endoplasm of the live parasites, cytoplasmic streaming was observed.

In the pyriform stages, vacuoles measuring 2.8–4.3 µm (neutral red-positive) (Figure 1D; Video S2) and refractive bodies measuring 0.9–1.8 µm were observed, and the latter seemed to be concentrated at the rounded end, posterior to the hyaline area (Figure 1B–D). The proliferative myxozoan stages showed intense exogenous budding by plasmotomy (Figure 1E–G and 1J–K). Thereby, the buds seemed to emerge actively from the “mother” parasite: The F-actin-rich ectoplasmic edge of still attached buds was found to occur on the opposite side than that of the “mother” parasite (Figure 1J–K), thus causing separation from it. Until complete separation, a cytoplasm bridge (approx. 6 µm length) was observed between separating stages (Figure 1K).

Parasites prepared for SEM showed a slightly different morphology than fresh material. Filopodia and the posterior, pointed cytoplasm extension were often difficult to distinguish (Figure 1F). However, the buds were clearly visible, and some of them showed a well defined demarcation line, a cytoplasmic constriction, at the point where they would eventually separate from the “mother” parasite (Figure 1E–F). In addition, using SEM, some small blebs (0.22–0.64 µm in diameter) were detected on the surface of the proliferating parasites (Figure 1E–G). Nile red-positive lipid droplets (1–2 µm in diameter) were very common in the larger pyriform parasites (Video S3) but not in the smallest ellipsoid stages.

**Figure 3:**
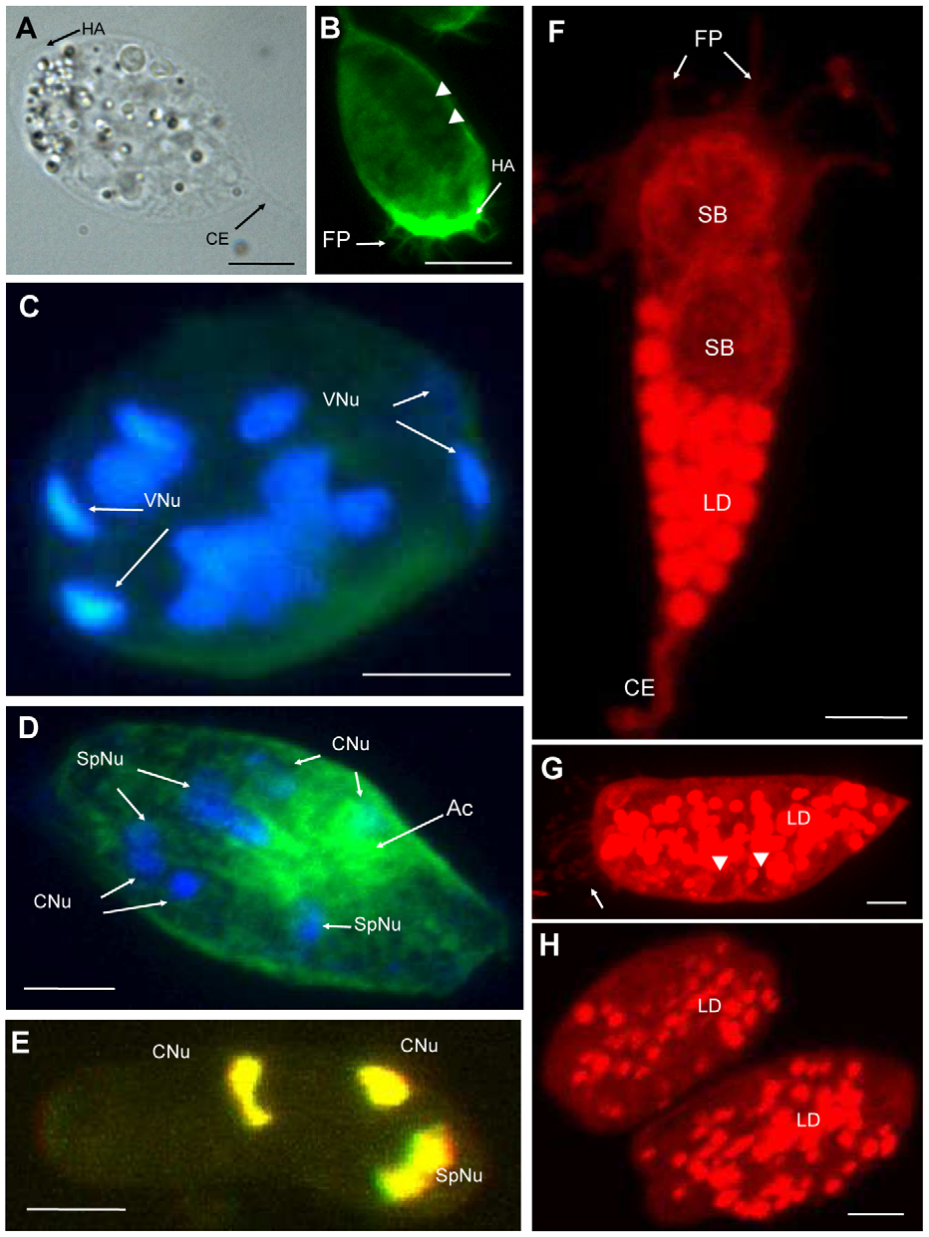
End of motility and the lipid droplets of *Ceratomyxa puntazzi* from the bile of *Diplodus puntazzo*. A: LM, B–H: CLSM. B: phalloidin stain, C–E: DAPI and phalloidin stains, F–H: Nile Red stain. A) Pseudoplasmodia with two almost mature spores. Hyaline area visible at rounded side of parasites and one cytoplasmic extension at posterior end. B) A early sporogonic stage, still pyriform, with a F-actin rich hyaline area and filopodia. Notice two polar capsules (arrow head) of the forming spores. C) Late pseudoplasmodium with two spores, where all nuclei, including the valvogenic ones are visible. D) Mature stage with two spores, where F-actin accumulation can be noted. Valvogenic nuclei have disintegrated. E) Mature spore liberated into the bile with two capsulogenic nuclei and two sporoplasm nuclei. Valve nuclei are absent. F) Pyriform stage with two early sporoblasts, abundant lipid droplets, filopodia and one larger posterior cytoplasmic extension. G) Almost mature stage showing abundant filopodia at rounded side (arrow) and abundant lipid droplets. Two polar capsules can be estimated (arrow head). H) Two mature stages harbouring two spores each, presenting smaller and randomly distributed lipid droplets than in early pseudoplasmodia. Abbreviations: HA: hyaline area; CE: cytoplasmic extension; VNu: Valvogenic nuclei; Ac: F-actin; SpNu: Sporoplasmogenic nuclei; CNu: Capsulogenic nuclei; FP: filopodia; SB: sporoblast; LD: Lipid droplets. Scale bar: A = 10 µm; B = 8 µm; C–H = 4 µm.

Sporogonic stages of *C. puntazzi* were represented by pseudoplasmodia developing two crescent-shaped spores (Figure 3A). Early sporogonic stages were pyriform and continued to show a high degree of activity and amoeboid-like movement as well as an F-actin-rich anterior edge (Figure 3B), as seen in proliferative stages. While more features of the mature spores became visible, the parasite stages showed less locomotive activity and were more rigid, due to the mechanical effect of the almost mature spores. As sporogenesis proceeded, F-actin was more dispersed and no longer concentrated at one extremity (Figure 3D). Filopodia were present throughout the initial stages of sporogony (Figure 3F–G) but had disappeared by the time spores neared maturity (Figure 3H). The pyriform shape was found to change to an oval one and lipid droplets were less abundant and had a more random distribution (Figure 3H) in mature pseudoplasmodia. In some mature pseudoplasmodia, more than one cytoplasmic extension could be observed (Video S4). Mature spores were liberated into the bile.

### Cellular composition and developmental sequence

#### Proliferative stages

**Figure 4:**
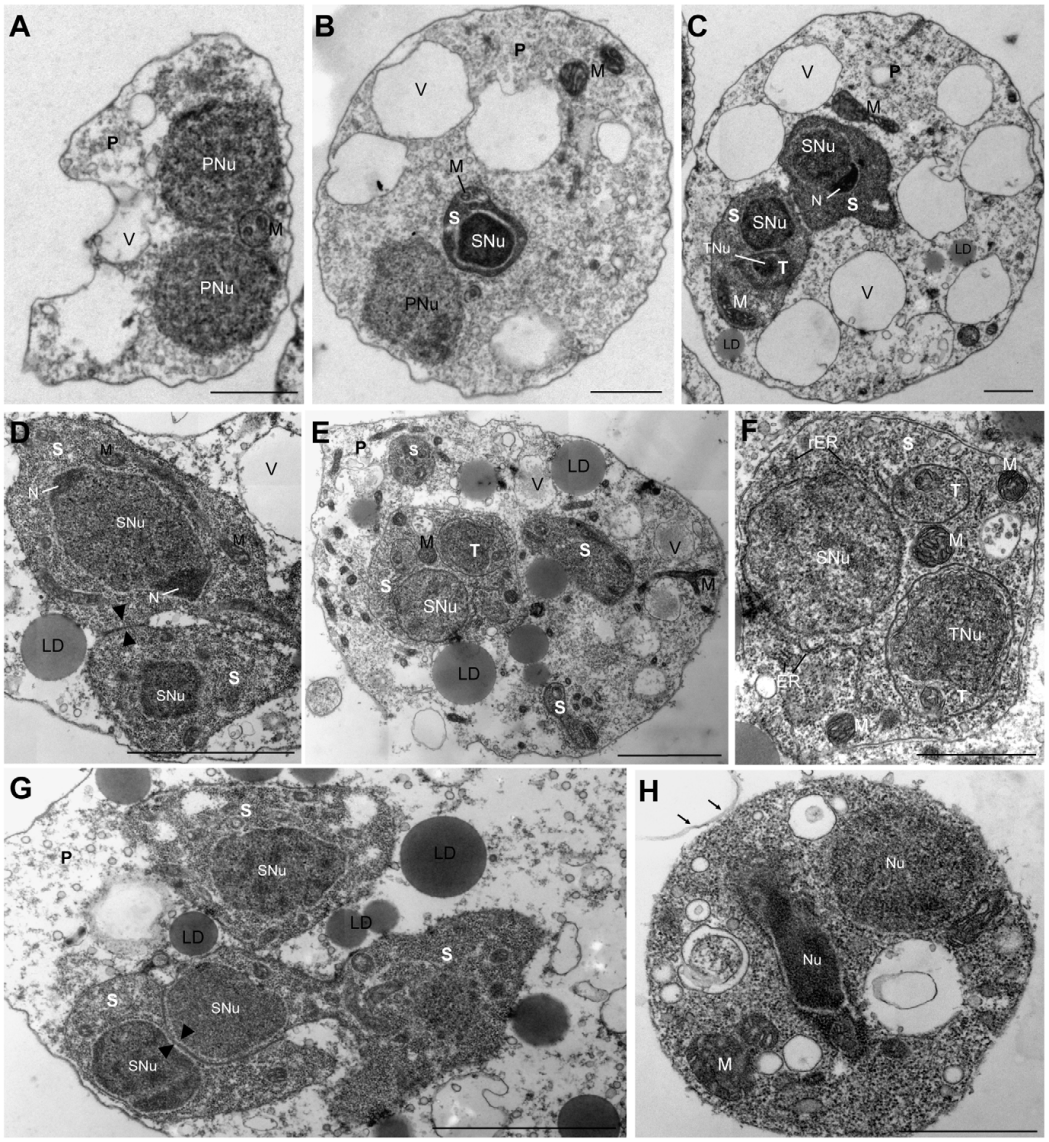
Pre-sporogonic proliferative development of *Ceratomyxa puntazzi* from the bile of *Diplodus puntazzo* (TEM). A) Early stage consisting of a P cell with two P cell nuclei. Notice the presence of vacuoles, mitochondria and absence of lipid droplets. B) Stage consisting of a P cell and a S cell with their respective nuclei. In the P cell, vacuoles are well defined and large mitochondria. In the S cell, small mitochondria are visible. C) Stage consisting in a P cell with two S cells, one of them with a forming tertiary cell (S-T doublet). Large mitochondria are present in the P cell and in the S cells. One of the S cell nuclei has an eccentric nucleoli. Notice the presence of small lipid droplets in the P cell. D) Detail of a stage showing two S cells and their nuclei, one of them with two eccentric nucleoli. Notice junction of the S cells (arrow head). Electron-dense lipid droplets where observed in the cytoplasm of the P cell and abundant mitochondria in the S cells. E) Large stage with several S cells and S-T doublet. Abundant electron-dense lipid droplets and mitochondria in the P cell. F) Detail of S-T doublet shown in Figure 4E, composed of an S cell and two T cells. Notice abundant rough endoplasmic reticulum in S cell cytoplasm. G) A stage with three S cells in a P cell. Notice cell junction of two S cells (arrow head), where partial engulfment was detected. H) Liberated cell doublet was observed, with a high electron dense cytoplasm, probably a S cell with a T cell in its cytoplasm. Note remnants of the P cell membrane (arrows). Abbreviations: P: primary cell; PNu: primary cell nucleus; M: mitochondria; S: secondary cell; SNu: secondary cell nucleus; V: vacuole; LD: lipid droplet; N: Nucleoli; T: tertiary cell; TNu: tertiary nucleus; rER: rough endoplasmic reticulum. Scale Bar: A–C = 1 µm; D–E = 2 µm; F = 1 µm; G = 2 µm; H = 1 µm.

TEM showed that the earliest, spherical stages observed in the lumen of the gall bladder represented a primary (P) cell containing one or two nuclei (Figure 4A), which occupied most of the cell lumen. Larger, pyriform stages with 1–2 nuclei in the P cell contained up to 6 secondary (S) cells (Figure 4B–C), and later, S cells were found to harbour 1–2 tertiary (T) cells (Figure 4C and 4E–F). The largest proliferative stages were found to contain 12 nuclei (Figure 1H). Commonly, units of 1–3 S cells, harbouring 0–2 T cells each (Figure 4F) were found to be separated from larger stages and to bud off (Figure 1E–F and 1J). Often, proliferation inside the different compartments would continue while the buds were still attached.

**Figure 5:**
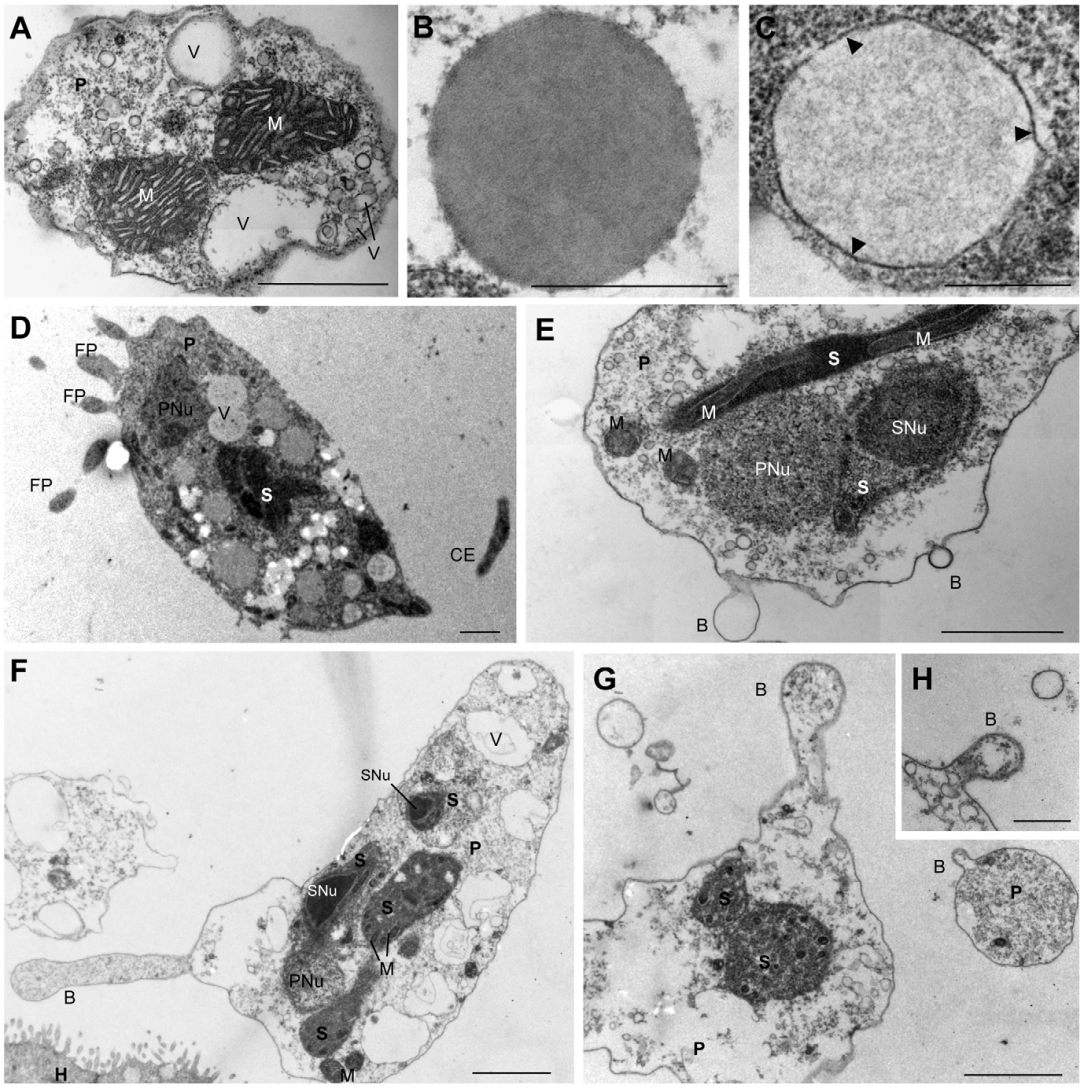
Pre-sporogonic proliferative development of *Ceratomyxa puntazzi* from the bile of *Diplodus puntazzo* (TEM). A) Two large mitochondria in the P cell with numerous well-developed cristae. B) Detail of electron-dense lipid droplet in a P cell. Notice the lack of a delimiting membrane. C) Detail of non electron-dense vacuole with a single membrane (arrow head) filled with granular material. D) Pyriform stage with filopodia at rounded side and a large cytoplasmic extension at posterior end. Notice vacuoles with granular material. E) Stage with blebs on the surface. Notice electron-dense cytoplasm of an S cell densely packed with ribosomes. F) Large stage close to the epithelium of the gall bladder, with a large bleb filled with the same material as the cytoplasm of the P cell. G) Stages with blebs. Notice the presence of material inside, similar to the P cytoplasm content. H) Detail of a bleb. Abbreviations: P: primary cell; PNu: primary cell nucleus; M: mitochondria; S: secondary cell; SNu: secondary cell nucleus; V: vacuole; FP: filopodia; CE: cytoplasmic extension; B: bleb; H: host epithelium. Scale Bar: A = 1 µm; B–C = 0.5 µm; D–E = 1 µm; F–G = 2 µm; H = 1 µm.

P cells had the largest nuclei and generally showed dispersed heterochromatin. The cytoplasm of the P cell was generally less electron-dense than that of S or T cells as it was less densely packed with ribosomes. The smallest stages, *i.e.* the ones harbouring 0–2 S cells, often contained very large mitochondria (0.9–1.2 µm length) with various pronounced cristae (Figure 5A). The number of mitochondria increased as the P cells grew (Figure 4E) but their size was considerably smaller (0.2–0.8 µm length) in larger parasites. Lipid droplets (Figure 5B) in the P cell were absent or few and small in the youngest proliferative stages (Figure 4A–C) but numerous and larger in multicellular stages (Figure 4E). Lipid droplets were rarely observed in the cytoplasm of S or T cells. The P cell of proliferative stages of all sizes had a large number of non electron-dense vacuoles (Figure 5C). These had a single membrane and were of variable size, measuring 0.9–2.3 µm in diameter (Figure 5A), sometimes filled with granular material (Figure 5D).

The cytoplasm of S cells contained mitochondria, abundant ribosomes and rough endoplasmic reticulum (Figure 4F). Mitochondria of S cells were normally smaller (0.2–0.4 µm in length) than mitochondria of early P cells. Between some S cells, prominent cell junctions were observed (Figure 4D and 4G). In some cases, partial engulfment was detected between attached S cells (Figure 4G). Prominent eccentric nucleoli were sometimes present in the nuclei of S cells (Figure 4C–D). A liberated cell doublet was observed, with a highly electron dense cytoplasm, probably representing an S cell harbouring a T cell in its cytoplasm, and with remnants of the P cell membrane attached (Figure 4H).

Generally, the T cell cytoplasm was even more densely packed with ribosomes than that of S cells, and some small mitochondria were also present (Figure 4F).

The cytoplasmic extensions corresponding to the rhizoid filopodia at the anterior end and the rigid posterior cytoplasm extension were also observed in TEM sections (Figure 5D). Small blebs were identified on the surface of the P cell by SEM (Figure 1E–G) and were also detected in TEM sections (Figure 5E–H). Their content was either transparent (Figure 5E) or presented the same structure as the cytoplasm of the P cells (Figure 5F–H).

#### Sporogonic stages

**Figure 6:**
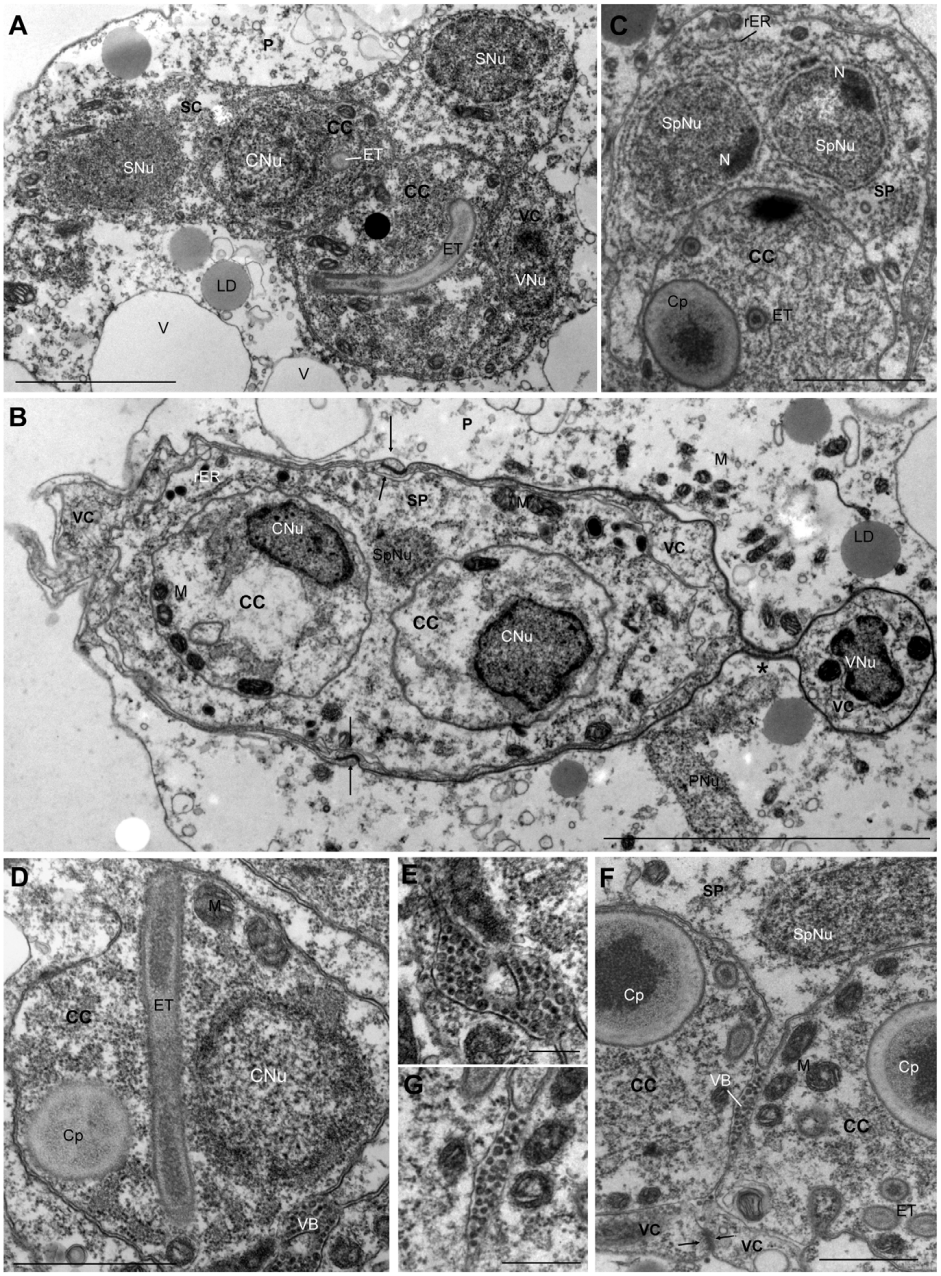
Sporogenesis of *Ceratomyxa puntazzi* from the bile of *Diplodus puntazzo* (TEM). A) Initial sporoblast with two capsulogenic cells developing the external tube. Both capsulogenic cells are enveloped by a sporoplasmogenic cell harbouring two sporoplasmic nuclei. Laterally, a valvogenic cell and its nucleus. Lipid droplets and vacuoles are abundant in the cytoplasm of the P cell. B) Sporoblast with two capsulogenic cells, a sporoplasmogenic cell with two nuclei and two valvogenic cells. The nucleus of the valvogenic cell is connected by a cytoplasmic bridge (*). Notice formation of suture (arrows). C) Detail of a sporoblast, showing the binucleate sporoplasm, with two eccentric nucleoli. Abundant rough endoplasmic reticulum is present in the cytoplasm of the sporoplasmogenic cell. D) Capsulogenic cell with a prominent external tube and a capsular primordium. Note vesicular body associated to the membrane of the capsulogenic cell. E) Detail of the vesicular body of Figure 6D. F) Detail of a sporoblast with vesicular body between the membranes of the two capsulogenic cells and the sporoplasmogenic cell. Suture forming between the two valvogenic cells (arrows). G) Detail of vesicular body shown in Figure 6F. Abbreviations: P: primary cell; PNu: primary nucleus; CC: capsulogenic cell; CNu: capsulogenic nucleus; SP: sporoplasmogenic cell; SpNu: sporoplasmogenic nuclei; VC: valvogenic cell; VNu: Valvogenic nucleus; ET: external tube; rER: rough endoplasmic reticulum; LD: lipid droplets; M: mitochondria; V: vacuole; Cp: capsular primordium; N: nucleoli; VB: vesicular body. Scale Bar: A = 2 µm; B = 5 µm; C = 2 µm; D = 1 µm; E = 0.2 µm; F–G = 1 µm.

Sporogony was initiated from a pyriform P cell (the pseudoplasmodium) containing 4 S cells. Further division led to the formation of 8 S cells and 4 T cells. These would separate in two pairs of 4 S cells and 2 T cells, each forming a spore. Valve cells were formed from S cells, whereas capsulogenic cells were formed from T cells whose enveloping S cells were found to form the sporoplasm (Figure 6A–B). In the beginning, the sporoplasmogenic cells would take up most of the space inside the developing spore. More mature capsulogenic cells were found to be attached to the valve cells (Figure 6A).

**Figure 7:**
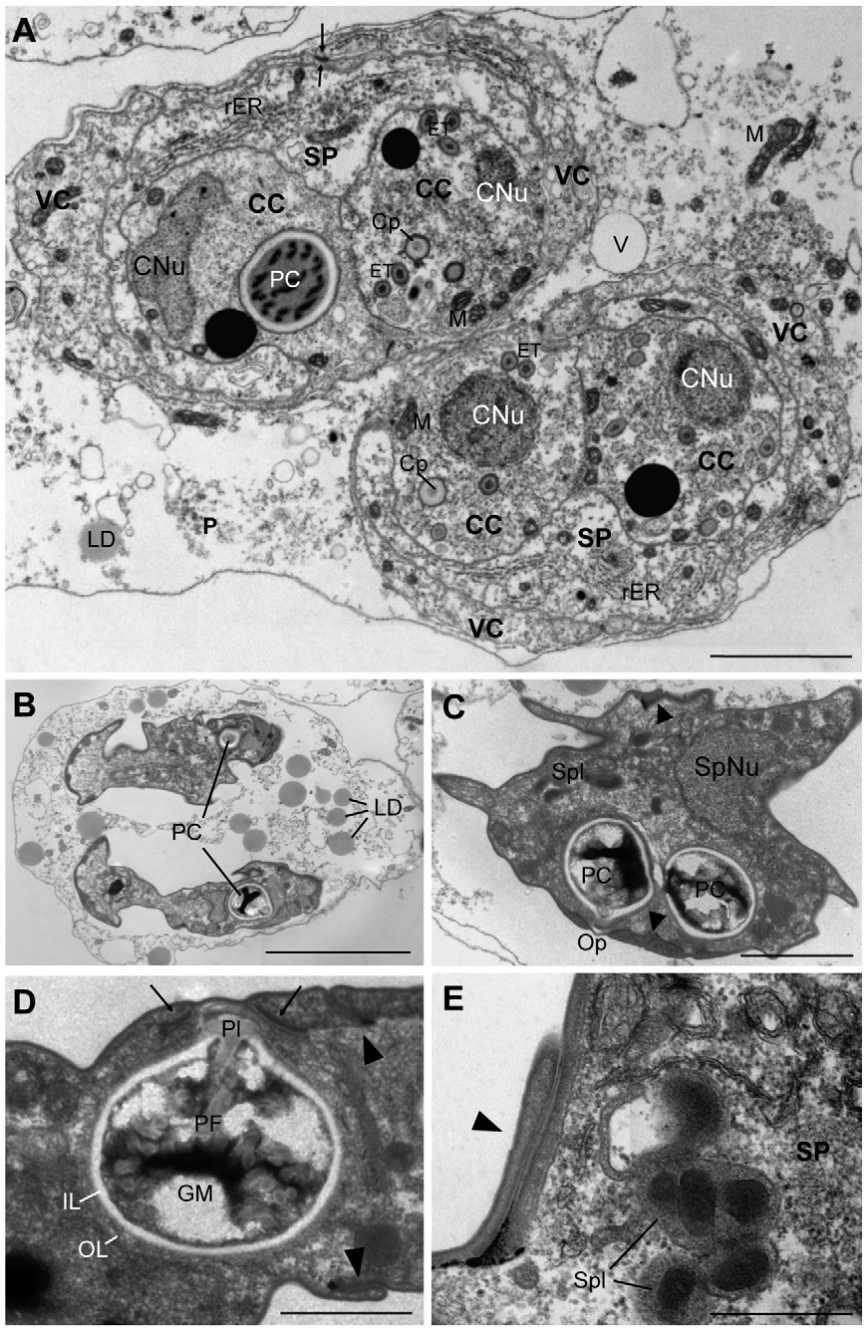
Sporogenesis of *Ceratomyxa puntazzi* from the bile of *Diplodus puntazzo* (TEM). A) Pseudoplasmodium harbouring two sporoblasts, with capsulogenic cells in different stages of maturation. One capsulogenic cell has a mature polar capsule, where sections of the coiled, twisted polar filament can be observed. The other three capsulogenic cells show an external tube and capsular primordium in their cytoplasm. Note abundant rough endoplasmic reticulum in the cytoplasm of the sporoplasmogenic cell. Formation of suture line between valvogenic cells can be observed (arrows). B) Two mature spores inside a P cell. Abundant lipid droplets are present in the degenerated P cell. C) Mature spore with two polar capsules, the opening for the extrusion of the polar filament, the suture (arrow head), one sporoplasm nucleus and sporoplasmosomes present in the sporoplasm cytoplasm. D) Detail of a mature polar capsule with twisted appearance of polar filament and capsular plug; polar capsule with an outer electron-dense layer and an inner electron-lucent layer. Apical junctions of the mature spore, surrounded by fibrous material between the capsulogenic cell and the valve cell (arrows). Suture between valves (arrow head). E) Detail of a mature spore: desmosome-like junction of the suture (arrow head) and sporoplasmosomes in sporoplasm cytoplasm. Abbreviations: CC: capsulogenic cell; CNu: capsulogenic nucleus; SP: sporoplasmogenic/sporoplasm cell; SpNu: sporoplasmogenic/sporoplasm nucleus; VC: valvogenic cell; PC: polar capsule; Cp: capsular primordium; ET: external tube; M: mitochondria; rER: rough endoplasmic reticulum; LD: lipid droplets; Spl: sporoplasmosomes; Op: opening for the extrusion of the polar filament; PF: polar filament; Pl: capsular plug; IL: inner electron-lucent layer; OL: outer electron-dense layer; GM: granular matrix. Scale Bar: A = 2 µm; B = 5 µm; C = 2 µm; D = 1 µm; E = 0.5 µm.

The pseudoplasmodia (P cells) were electron-lucent and, in the early stages of sporogony, the P cells contained a large number of mitochondria, lipid droplets and vacuoles (Figure 6A–B), but as spore formation proceeded, the P cell cytoplasm appeared less defined and less electron-dense, still containing lipid droplets but having less vacuoles (Figure 7A–B). At the end of sporogenesis, the P cell cytoplasm had degenerated and contained hardly any organelles apart from abundant lipid droplets (Figure 7B).

During sporogony, the valvogenic cells soon occupied an external position enveloping all other sporogonic cells (Figure 3C, 6A and 7A). In one case, during early sporogony, the cytoplasm of a valvogenic cell showed a constriction connecting an area containing the nucleus of one valvogenic cell with the remainder of the valvogenic cell and all other spore-forming cells (Figure 6B). During spore maturation, the cytoplasm of the valvogenic cells became progressively thinner. The two valvogenic cells joined to form the suture (Figure 6F and 7A), in which the two cells overlapped and formed a desmosome-like junction (Figure 7C–E). Mature valve cells formed only a thin line surrounding the other sporogonic cells and the valve cell nuclei had disintegrated (Figure 3E).

Capsulogenic cells developed a capsular primordium with an external tube in the cytoplasm (Figure 6A and 6D). As capsulogenesis advanced, the capsular primordium and the external tube grew. Later on, the polar filament was found to condense (Figure 7A and 7D) and coil up inside the polar capsule, forming five coils. The mature polar filament was typically twisted and lay in a granular matrix (Figure 7D). Polar capsules had an outer electron-dense layer and an inner electron-lucent layer (Figure 7D). At the end of capsulogenesis, the polar capsules acquired a pointed tip where the plug for polar filament discharge was located (Figure 7C–D). Next to the plug, the junctions between the capsulogenic cells and the valve cells were surrounded by fibrous material (Figure 7D). Capsulogenesis was not synchronised between the four capsulogenic cells of a pseudoplasmodium (Figure 7A).

The two sporoplasmogenic cells (Figure 6A) formed a single binucleate sporoplasm in mature spores (Figure 6C). The nuclei of the sporoplasmogenic cells contained heterochromatin, only in mature stages one eccentric nucleoli was detected in each nucleus (Figure 6C). In contrast to the P cell, the cytoplasm of sporoplasmogenic cells showed abundant rough endoplasmic reticulum (Figure 7A). In the cytoplasm and between the membranes of the two capsulogenic cells, an organized “vesicular body” was observed (Figure 6D–G). The “vesicles” ranged from 32 to 60 nm in diameter: in some cases small electron-dense dots were present inside the “vesicles” (Figure 6E and 6G). Sporoplasmosomes and membrane bound structures appeared in the cytoplasm of the sporoplasm close to the end of sporogenesis (Figure 7C and 7E).

**Figure 8:**
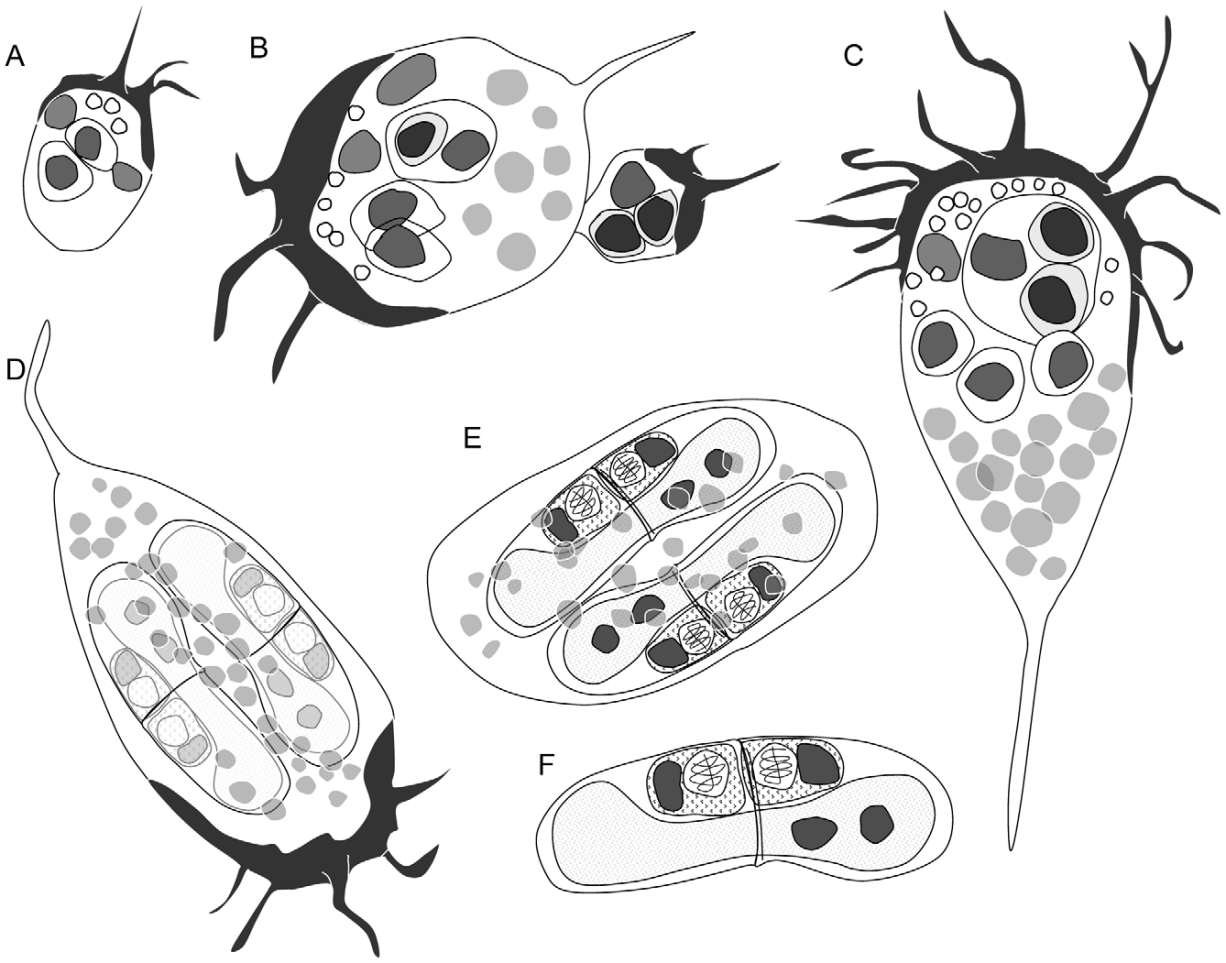
Diagrams of some representative developmental stages of *Ceratomyxa puntazzi* in the bile of *Diplodus puntazzo*. A) Early, active ellipsoidal stage with a few filopodia at anterior part, showing a primary cell with two primary nuclei and two secondary cells with a nucleus each, and refractive granules. B) Budding of a small stage from the “mother” parasite. Both stages show accumulation of F-actin in opposite poles allowing separation. “Daughter” parasite shows our suggestion of a secondary-tertiary cell doublets as proliferative stages. C) Active, pyriform stage, with many filopodia, at anterior part, some of them ramified. This stage possesses a primary cell with a primary nucleus, three single secondary cells and a secondary cell with two tertiary cells, abundant lipid droplets and refractive granules. D) Sporogonic stage with two forming spores, still showing motility and abundant lipid droplets. E) Sporogonic stage close to the end of spore development, with loss of motility and reduced size and number of lipid droplets. F) Mature spore with a binucleate sporoplasm, showing two capsulogenic cells with their nuclei and two polar capsules.

In order to summarize the results obtained from the combination of different microscopic techniques and stains, we produced diagrams representing different developmental stages of *C. puntazzi* (Figure 8).

## Discussion

### CLSM: an unexploited tool for myxozoans

As the present study shows, the combination of light microscopy, scanning and transmission electron microscopy and three-dimensional confocal laser microscopy, successfully contributed novel information on the structure and morphology of ceratomyxid parasite stages in the bile, and provided unique insights into parasite composition, cell motility and cytokinesis in myxozoans, which had not previously been studied. Thereby, CLSM presents itself as a poorly exploited but extremely useful tool for exploring the three-dimensional morphology of the parasites as well as for determining the presence and location of certain cellular components. By using DAPI nucleic acid stain in CLSM in combination with TEM it was possible to determine the number of nuclei in each parasite stage and during budding. The occurrence of pre-sporogonic proliferation and spore formation at the same site allows parasite multiplication directly prior to spore formation and thus increases the parasite's number and chance of infecting the invertebrate host considerably. The co-location of proliferative and sporogonic cycles in the same organ has also been reported for other *Ceratomyxa* spp. [23], [25]–[26], [32]–[34].

### F-actin appears to be a motility effector in myxozoans

The use of phalloidin, for the first time in myxozoan proliferative stages, suggests that F-actin is an important effector of cell motility in myxozoans. In *C. puntazzi*, and possibly in other myxozoans inhabiting liquid-filled body cavities, motility of the parasites is achieved by a highly active, polarized F-actin rich cytoplasm area, the ectoplasm, which is located at the anterior pole of the parasite. This area represents a cell membrane protrusion or lamellipodium which further produces filopodia. The filopodia showed a very high degree of mobility and *de novo* creation and re-absorption by the P cell. Thereby, they were found to extend forward in the direction of swimming and then moved postero-laterally, thus facilitating directional movement. The involvement and function of the structures and F-actin have not previously been studied or documented in the Myxozoa. However, previously, *Ceratomyxa* spp. have been described to possess cytoplasmic extensions [26], [34]–[35]. However, they have been related predominantly to anchorage in the host epithelium and to a potential increase in the absorptive surface which is in contact with host cells [36]–[39]. In myxozoans, pinocytosis has been observed as a means of energy transfer from host to parasite, with the P cell taking up material from the surrounding host cell, but also from P cells to S cells [40]. In the case of *C. puntazzi*, none of the cytoplasmic extensions detected were found to be in contact with the epithelium of the gall bladder, and all parasites were found to be in suspension in the bile. Based on this observation as well as on the high activity level of the filopodia in *C. puntazzi*, it is strongly suggested that they have a locomotive rather than an anchoring or energy providing function. However, it remains unclear, if the parasite's energy to fabricate new cellular components can come exclusively from the bile.

Information on the occurrence and distribution of effectors of cell motility in myxozoans is scarce. F-actin has been previously described to be present in the stinging tube of the polar capsules and in the cytoplasm of the amoeboid sporoplasm of actinosporean spores of *Myxobolus pseudodispar* [41]. However, this is the first report of the presence of an F-actin rich cytoskeleton in proliferating stages, confirming the active role of these stages. Almost mature pseudoplasmodia showed much more dispersed F-actin and less locomotive activity and flexibility. The loss of F-actin may reflect the end of the active swimming period and may cause sinking and thus allow expulsion from the gall bladder via the common bile duct, resulting in the release of spores via the intestine.

### Polarized F-actin distribution allows budding

The present study further demonstrates for the first time, that F-actin seems to be of major importance in the process of budding. Budding has previously been observed in many members of the genus *Ceratomyxa*, *e.g.* in *Ceratomyxa appendiculata*, *Ceratomyxa herouardi*, *Ceratomyxa blennius* and *Ceratomyxa protopsettae* [23], [26], [32], [35]. However, a mechanism resulting in the separation of newly formed “daughter” parasites has so far not been suggested. In contrast to the majority of eukaryotic cell divisions, in the present study, cytokinesis A, the purse string model, was not observed, as an F-actin rich contractile ring was absent during the budding process. Separating buds were always found to “head” away from the “mother” parasite, demonstrating polarization of the F-actin cytoskeleton at the opposite end of its attachment to the “mother” parasite, thus resulting in the active separation of buds. Cytokinesis B and C are modes of cell division which seem to be carried out by passive constriction and active protrusion [14] as observed in the present case, and they are considered primitive modes of cytokinesis, due to their lower demand of proteins and functionality [11]. Cytokinesis B and C are defined as substrate dependent. However, attachment of *C. puntazzi* to the gall bladder epithelium was not observed in the present study, although widely reported in other *Ceratomyxa* spp. [42]–[44]. The type of cytokinesis observed for the Myxozoa in the present study does not match all characteristics of the existing models type B or C and it is still unclear if it can be ascribed to one of them, however, it is clear that it excludes the formation of a myosin II-dependent contractile ring (Cytokinesis type A). It thus differs from the general mode of cell division in eukaryotes. Further research is needed to confirm the active role of F-actin in myxozoan motility and budding by using anti-microfilament drugs. Furthermore, it would be important to determine whether other proteins are implicated in the cytokinesis of myxozoans to be able to determine their evolutionary relatedness. Thereby, the related proteins may be of particular interest for phylogenetic studies as the exact origin of the myxozoans is still unclear, despite their recent adscription to the Cnidaria [45]–[47].

### Composition of “daughter” parasites: The fate of the P cell

Plasmotomy appears to be the process leading to the division of the P cell during exogenous budding as observed in SEM of *C. puntazzi*. Plasmotomy has also been reported from other marine coelozoic myxosporeans [23], [32], [48]. However, despite the large number of TEM sections examined, the exact composition of “daughter” parasites remains unknown. “Daughter” parasites contained 1–3 S cells, harbouring 0–2 T cells each, but it is unclear whether they contained a P cell nucleus, as it was not observed in the buds. However, in the present study, the P cells of *C. puntazzi* appeared to be rather inactive with no more than 2 nuclei present in any parasite stage, at any given time, when compared with S and T cells, which multiplied frequently and were present in larger numbers. This suggests that “daughter” parasites do not contain a P cell nucleus but only part of the P cell cytoplasm, which probably disintegrates or is being reabsorbed resulting in the liberation of S cells enveloping T cells. Support for this idea is found in the observation that some small stages of one cell enveloping another one were found to have a very electron-dense cytoplasm (Figure 4H), similar to that of S and T cells found within the P cell (Figure 4C and 4E) and remnants of a cell membrane (presumably that of the P cell) were attached to it. The formation of a demarcation line between buds and “mother” plasmodium seems to indicate that the release of buds does not result in the disintegration of the P cell of the “mother” plasmodium, unlike in proliferative blood stages of myxozoans [49]. Despite the large number of sections examined, the mechanism producing S or T cells in *C. puntazzi* could not be determined with certainty, however S cells engulfing other S cells were observed. Engulfment of one cell by another to form an S-T cell doublet was clearly demonstrated for *Ceratomyxa sparusaurati* [25] and supports the idea that T cells are generally produced by engulfment [50], however, this process has never been observed in P cells (resulting in the formation of S cells), and further ultrastructural studies of sequential sections is necessary to clarify whether engulfment is the only process of endogenous cell formation in myxozoans, as stated by Morris [50].

Cell junctions between S cells have previously been observed in other myxozoans [34], [42]. S cell junctions might make it easier for one S cell to envelop another S cell, and this might explain why they were observed frequently. It is likely that, after mitosis, S cells remain somehow attached, establishing the observed cell junctions.

### The role of the lipid droplets

Nile Red had not previously been used for CLSM in myxozoans, but allowed for the visualisation of the distribution of lipid droplets during parasite development. Lipid droplets were extremely common in medium-sized pyriform stages and less common in the smallest, ellipsoid stages or in late sporogonic stages. Lipid droplets were almost exclusively present in the P cell. This was also observed in *Enteromyxum* spp. [22], [51]. However, lipid droplets have been observed in S and T cells, *e.g.* in sporoplasmogenic cells [52]–[53] or in capsulogenic cells [54]–[55]. In the case of *C. puntazzi*, the high concentration of lipid droplets in the P cell and the presence of considerably more rough endoplasmic reticulum and ribosomes in the S cells than in the P cell, may be indicative of different cell functions. In this respect, the non-dividing P cell may serve as a “container” and potential energy supplier for S and T cells, which divide frequently and are actively synthesizing cellular components. Thus, the lipid droplets might represent energy reserves for parasite proliferation and sporogony. At the same time, the lipid droplets might have some hydrostatic function, contributing to keeping the parasites neutrally buoyant in the bile. Surface enlargement by the formation of pseudopodia, their mobility and the presence of small blebs on the surface of the parasite's P cell might further increase hydrostasis and buoyancy.

### Further ultrastructural observations

Many ultrastructural details of *C. puntazzi* are similar to that of other Myxozoa, however, there are some structures that differ or are extraordinary and these are discussed briefly in the following section:

The P cells of *C. puntazzi* presented a very large number of vacuoles, sometimes filled with granular material. Their function is unclear. In the case of *Enteromyxum scophthalmi*, a nutritional role was ascribed to vacuoles in the P cell, with transport of nutrients to the S cells [22]. However, as the composition of the vacuoles is unknown, an excretory function could also be assigned. Additionally, the bile is rich in salts and vacuoles of the P cell could have an osmotic function, offsetting the loss of water of the parasite.

Shrinkage of the stages during fixation and staining may explain the discrepancy in the size of the vacuoles of the P cell between fresh LM samples and fixed TEM samples. A similar phenomenon was reported for myxozoan spores [56]–[57]. In a similar way, parasites prepared for SEM showed a slightly different morphology than fresh material, possibly due to fixation. Future studies will include rapid-freeze cryo-microscopy to circumvent these problems.

Mitochondria were considerably smaller in S cells than in early P cells, where extremely large mitochondria were observed. This could be related to the high energy production required for the motility of the highly active parasite stages, with locomotive actions likely to be conducted exclusively by the P cell.

A vesicular body was observed in the cytoplasm of the sporoplasmogenic cell, and it was found to be associated with the two capsulogenic cells, often demonstrating the only structure located between the membranes of the capsulogenic cells. A similar structure, described as aggregates of microtubules, was described in the capsulogenic cells of *Ceratomyxa tenuispora* [58], however, the diameter of the microtubules was slightly smaller (25 nm) than that of the vesicles (32–60 nm) of the vesicular body in *C. puntazzi*. Furthermore, both distribution and morphology were different. In *Polysporoplasma mugilis* from *Liza aurata*, icosahedral virus-like particles measuring 18–20 nm were observed [59]. These virus-like particles and the vesicular body of *C. puntazzi* share similarities: both structures represent a cluster of small vesicles surrounded by membranes, with an electron-dense core and are associated with the capsulogenic cell. However, the vesicular body in *C. puntazzi* differed considerably with regard to vesicle size, lacked icosahedral shape and was not associated with pathological effects. The vesicular body observed in the present study was previously reported in *Unicapsula pflugfelderi* where vesicles measured 40–60 nm. In *U. pflugfelderi* the vesicular body was also found in close vicinity to developing polar capsules, and a secretory function was suggested [60]. Due to its location and its vesicular structure it is suggested that the presence and function of the vesicular body is related to capsulogenesis, possibly secreting carbohydrates or other substances required for polar capsule formation.

To conclude, we demonstrate here that the combination of a three-dimensional microscopy method applying different specific stains together with the details of the cellular ultrastructure of the different parasite stages is the best approach to capture and understand the developmental cycles of myxozoan parasites, which are still poorly understood, due to their extremely reduced size and the lack of information on the basic principles of cell-in-cell formation. The material from the present study and comparison with existing reports showed that the development of *Ceratomyxa* spp. in the bile seems to follow a sequence which includes pre-sporogonic proliferation by budding and spore formation. Most importantly, previously undescribed details on the concentration of F-actin in certain cytoplasmic areas and the formation of active cytoplasmic extensions provided a first insight into the fascinating locomotive behaviour of myxozoans and the mechanisms of cytokinesis underlying the unique process of budding, which were found to differ from the general eukaryotic model. The knowledge of the presence of F-actin in myxozoan stages might contribute to design antiparasitic drugs that target F-actin affecting actin dynamics and cell motility, as some chemotherapeutics have been reported to perform at this level [61]. The use of other specific stains in CSLM is strongly encouraged as they are likely to provide further information on these processes and on the distribution and function of other important cellular components. Particularly exciting prospects are presented by the possibility to use CLSM in combination with vital stains and to observe the aforementioned processes *in vivo*.

## Materials and Methods

No ethic statement is required for this study. Fish handling was carried out according to the Spanish legislation (Real Decreto 1201/2005) and the Valencian regional legislation (Decreto 13/2007) for the protection of animals used for experimental and other scientific purposes. All animal work was approved by the ethics committee for animal welfare of University of Valencia, Valencia, Spain (license number: A1312449905843).

### Fish and parasite collection

Between January 2008 and June 2010, 81 specimens of sharpsnout seabream *D. puntazzo* (6–29 cm total length; 3.75–448.6 g) were obtained from San Pedro del Pinatar, Mar Menor, Murcia (Mediterranean, Spain), where they had been caught by netting. Fish were transported live to the aquaria facilities at the University of Valencia. For study of the myxozoan development in the fish, fish were euthanized by neural pithing. Whole infected gall bladders as well as parasite stages in freshly collected bile were analysed. Parasites were observed *in vivo*, as well as after fixation and differential staining, using a variety of microscopic techniques to obtain the most comprehensive information on the morphology and development of *C. puntazzi* in the gall bladder of *D. puntazzo*.

### Microscopy

#### Light microscopy (LM)

Several microliters of fresh bile were pipetted onto microscopic slides, coverslipped and observed by LM using Nomarski's differential interference contrast on a Leica DMR microscope under 100× objective lens. Digital images of live material were taken with a Leica DC300 (Leica Microsystems Ltd.). Using the computer software UTHSCSA ImageTool Version 3.0 for Windows (The University of Texas Health Science Center at San Antonio, Texas, USA), measurements were taken on digital images and in relation to images taken of a graticule of defined length. All measures are maximum length and width.

Videos of live parasites were recorded with a Leica DFC295 (Leica Microsystems Ltd.) mounted on the same microscope. In some cases, live parasites were stained with 1% neutral red. The videos were edited with ArcSoft ShowBiz® DVD 2 (ArcSoft Inc, USA).

#### Scanning electron microscopy (SEM)

Ethanol-washed and 0.1% poly-D-lysine coated slides were incubated with bile, containing different parasite stages, which were left to settle onto the coated surface for 30 min. The parasites were then fixed for 30 min on the coverslips using 2.5% glutaraldehyde in 0.1 M phosphate buffer (pH 7.4). After rinsing in PBS (2×15 min) the parasites on the coverslip were post-fixed with 1% osmium tetroxide in 0.1 M phosphate buffer for 30 min. Coverslips were then washed for 15 min in distilled water, dehydrated in an ascending alcohol series and critical-point dried. Thereafter, the coverslips were mounted on stubs, gold sputtered-coated and examined with an FeG-SEM Hitachi S4100 electron microscope (Hitachi High Technologies Co LTD, Tokyo, Japan).

#### Transmission electron microscopy (TEM)

Infected gall bladders were fixed in 2.5% glutaraldehyde in 0.1 M PBS (pH 7.4) for several days. Once transferred to the fixative, the gall bladder walls were penetrated using a syringe with a fine needle in order to allow immediate access of the fixative to the parasite stages. Fixation occurred over several days. Following several washes with PBS, the gall bladders were post-fixed with 1% osmium tetraoxide in 0.1 M PBS and washed again with distilled water. Gall bladders were transferred to 2% uranyl acetate in 30% acetone and incubated for 1 hour in the dark. Thereafter, they were dehydrated in an ascending acetone series and transferred into ALV resin, in which they were embedded and left to polymerise for 16 hours at 65°C. Ultrathin sections were cut and mounted on grids. Grids were examined in a Tecnai™ G2 Spirit BioTWIN (FEI Company, Oregon, USA) or a JEM 100B (JEOL Ltd, Peabody, MA, USA) transmission electron microscope.

#### Confocal laser scanning microscopy (CLSM)

For CLSM, 4% formalin fixed samples of infected bile in 0.1 M PBS were left to settle onto 0.1% poly-D-lysine coated slides for 30 min. The parasites were stained with the following differential dyes: 1. Nile Red (7-diethylamino-3, 4-benzophenoxazine-2-one), which is lipophilic and stains intracellular lipid droplets, 2. Phalloidin (Alexa Fluor® 488 phalloidin, Invitrogen), which binds specifically at the interface between the subunits of F(ilamentous)-actin, which forms the cytoskeleton and functions as an important mediator of cell motility, and 3. DAPI (4,6-diamidino-2-phenylindole, dilactate; Molecular probes), which binds to DNA. Nile Red stock solution (0.5 mg mL^−1^ in acetone) was diluted by mixing 1.7 mL of the stock solution with 50 mL of a 75∶25 glycerol∶water mixture. Nile red staining was conducted for 30 min in the dark, thereafter the samples were washed with 0.1 M PBS. Phalloidin was applied at 2.5 µL 100 µL^−1^ in 0.1 M PBS and DAPI at 300 nM in ionised tap water. Parasites were stained for 30 min, and no post-staining rinses were conducted before viewing. Cover slips were placed over all the samples and sealed with nail varnish to prevent evaporation of the medium. All CLSM samples were examined using a Leica TCS SP2 AOBS confocal laser scanning microscope (Leica Microsystems AG, Wetzlar, Germany).

## Supporting Information

Video S1 **Motility pyriform stages.** Several pyriform stages showing active locomotion, with a hyaline area at round side with abundant active filopodia projected from the most anterior part to the posterior part of the hyaline area. Largest stages presented fewer motility activity. Notice the cytoplasmic streaming, the accumulation of refractive granules, large vacuoles and the long and rigid posterior cytoplasmic extension.
(MPG)

Video S2 **Neutral red stained active pyriform stage.** Notice the large vacuoles distributed throughout the body and the abundant refractive granules at round side.
(MPG)

Vídeo S3 **CLSM reconstruction of a Nile red stained pyriform stage.** Lipid droplets distribution and two sporoblasts located at the wider part of the body of the parasite can be observed. Notice the distribution of the filopodia at the round side.
(MPG)

Vídeo S4 **Active sporogonic stage with spores.** A sporogonic stage with two spores, showing less motility activity and more rigidity, due to the presence of the spores. Notice the presence of cytoplasmic extensions and some small filopodia moving.
(MPG)
